# Supplementary material for: Flight Phenology of Elasmopalpus lignosellus (Lepidoptera: Pyralidae) in the Northwest Florida Panhandle
Source: Insects. 2023 Apr 2;14(4):354. doi: 10.3390/insects14040354 (PMC10146345; doi:10.3390/insects14040354)
Supplement: Supplementary file 1 [file insects-14-00354-s001.zip › insects-2222821-supplementary.pdf]

insects

MDPI

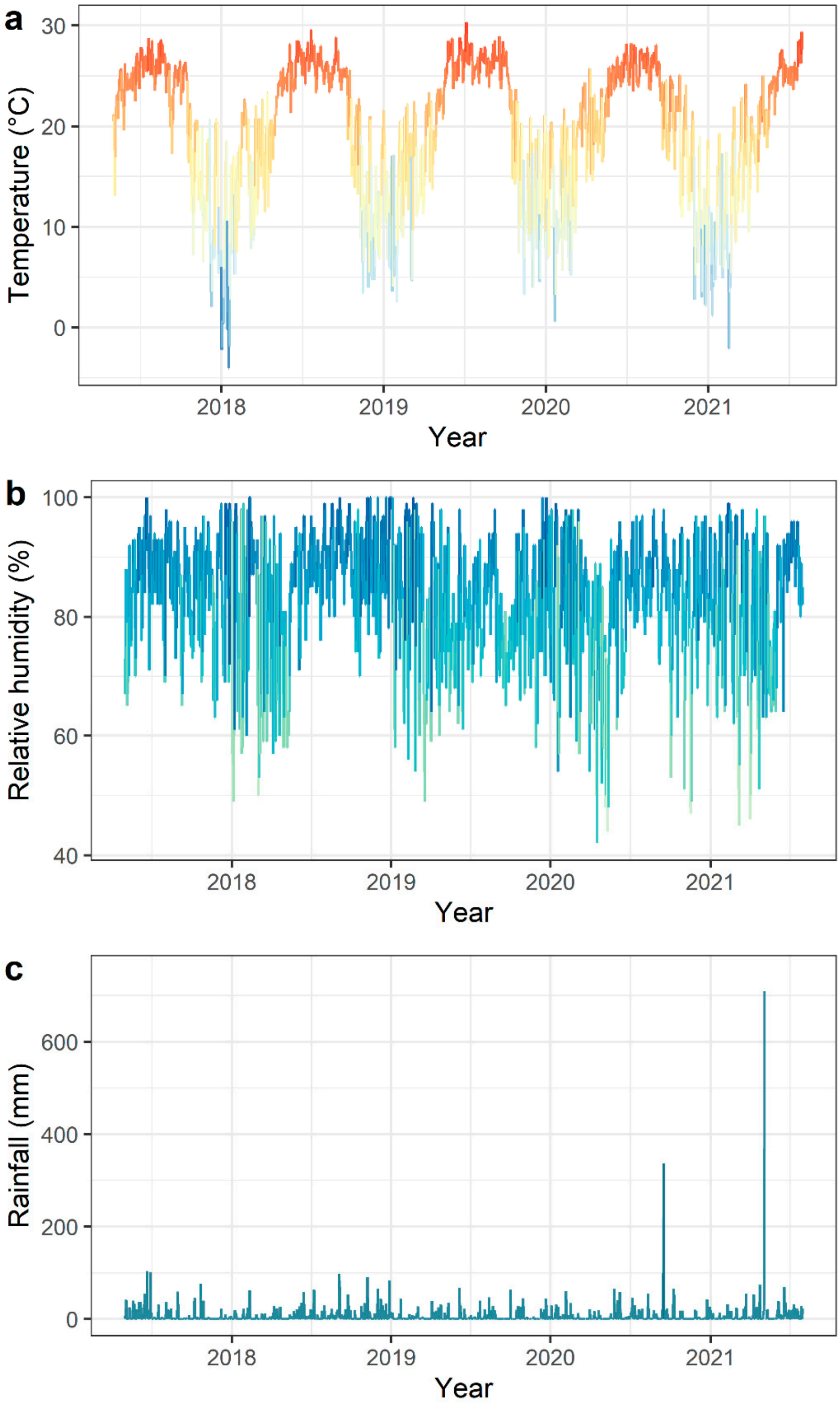

Figure S1. (a) Average daily temperature and (b) relative humidity, and (c) accumulated rainfall during the evaluation period, from Jul/2017 to Jun/2021, at Jay, Santa Rosa County, Florida.
